# Genome-Wide Identification of the *CIF* Gene Family and Protein Interaction with GSO1s Under the p-HBA-Induced Continuous Cropping Obstacle in *Pogostemon cablin*

**DOI:** 10.3390/ijms26041568

**Published:** 2025-02-13

**Authors:** Jieyun Fang, Siru Liu, Yating Su, Muhammad Zeeshan Ul Haq, Yougen Wu, Ya Liu, Xiuxia Ren

**Affiliations:** 1 School of Breeding and Multiplication (Sanya Institute of Breeding and Multiplication), School of Tropical Agriculture and Forestry, Hainan University, Sanya 572025, China; 2 Key Laboratory of Tropical Crops Nutrition of Hainan Province, South Subtropical Crops Research Institute, Chinese Academy of Tropical Agricultural Sciences, Zhanjiang 524091, China; 3 State Key Laboratory of Vegetable Biobreeding, Institute of Vegetables and Flowers, Chinese Academy of Agricultural Sciences, Beijing 100081, China

**Keywords:** *Pogostemon cablin*, Casparian strip, small peptide, stress, Alphafold

## Abstract

Casparian strip integrity factors (CIFs), which are tyrosine-sulfated small peptides, are crucial genes involved in the formation and regulation of the Casparian strip and play an important role in the regulation of plant stress response. In order to explore the evolution, characteristics, role, and function of *CIFs* in response to continuous cropping obstacles (CCOs), the bioinformatics and gene expression analysis of *CIF* genes in *Pogostemon cablin* was carried out by determining the phylogenetic relationship, chromosome location, gene structure, and RT–qPCR results. Results showed that a total of 12 *PatCIF* family genes were identified on 12 different chromosomes. Promoter prediction analysis revealed 16 different *cis*-regulatory elements. A systematic evolutionary study of 33 species indicates *CIF* family genes originated from Spermatophyta. Collinearity analysis revealed *P. cablin* shared 19 syntenic genes with *Solanum lycopersicum* and only 8 with *Oryza sativa*. Transcriptome analysis indicated that the expression of *PatCIF1–4* and *PatGSO1b/1c/1f* genes decreased under p-hydroxybenzoic acid treatment, and further RT–qPCR validation of four *PatCIF* genes was consistent with the results. AlphaFold prediction showed a protein interaction region between PatCIF1–4 mature peptide and PatGSO1b/1c/1f via the LRR domain, which provides a key binding surface for mature PatCIFs. This study offers a theoretical basis to investigate the roles of *PatCIFs* and *PatGSO1s* in CCOs and their protein interactions in *P. cablin*.

## 1. Introduction

*Pogostemon cablin* (Blanco) Benth., also called patchouli, is a perennial fragrant herb in the Lamiaceae family, originating from Southeast Asia [1,2]. It has been widely introduced and cultivated in many tropical and subtropical regions, including China, the Philippines, Indonesia, and Thailand [3]. In recent years, *P. cablin* has been proven to be rich in a variety of chemical components, including flavonoids, phenylpropanoids, various terpenoids (such as monoterpenes, sesquiterpenes, diterpenes, etc.), steroids, alkaloids, and fatty acids [4]. Among them, the essential oil (EO) of *P. cablin*, extracted from *P. cablin* shoots, is a crucial aromatic oil vital in the pharmaceutical, food, and cosmetic industries. The EO contains several sesquiterpenoids, with *P. cablin* alcohol as its main active ingredient [5,6]. As one of China’s Top Ten Southern Medicines, it has multiple pharmacological activities, such as anti-pathogenic, analgesia, anti-inflammatory, and regulation of gastrointestinal function [7]. So far, *P. cablin* research primarily focuses on identifying, extracting, and purifying medicinal components [8] or other secondary metabolisms [9].

The continuous cropping obstacle (CCO) is the phenomenon of reduced land productivity, and hence crop yield and quality, when the same or similar crops are grown consecutively on the same piece of land even under normal cultivation and management conditions [10,11]. This phenomenon is a common occurrence in agricultural cultivation and production processes. Currently, major crops, such as *Glycine max* [12], *Arachis hypogaea* [13], *Solanum lycopersicum* [14,15], and *Cucumis sativus* [16,17], have obvious problems of succession disorder. Noticeably, 70% of Chinese herbal medicines seriously suffered from CCO, such as *P. cabin* [18], *Panax ginseng* [19], *Rehmannia glutinosa* [20], *P. notoginseng* [21], *P. quinquefolius* [22], *Atractylodes lancea* [23], and *Pinellia ternata* [24]. For instance, CCOs seriously affect the yield and quality of *P. cablin*, limiting its industrial development [25]. Three fundamental causes allegedly causing CCOs of *P. cablin* are the deterioration of soil physical and chemical qualities, the accumulation of allelochemicals, and the loss of microbial community balance [26]. P-Hydroxybenzoic acid (p-HBA) is a key autotoxin that contributes to the development of *P. cablin* CCO, among the factors that influence it [27]. However, how does this chemical cause *P. cablin* CCOs, what kinds of genes play regulatory roles, and which pathways (signaling) are implicated in this practice? These questions require further study and in-depth exploration.

The Casparian strip is a specialized cell wall structure that surrounds the radial and transverse walls of endodermal cells, which contains the polymeric molecules lignin and suberin [28,29]. As a selective barrier, it effectively screens, blocks, and prevents unwanted ions or macromolecules from diffusing into the vascular system [30]. Casparian strip formation and integrity are regulated by multiple genes and signaling pathways, including CIF 1/2 [31], leucine receptor kinase (GSO1/SGN3) [32], enhanced suberin 1 (ESB1) [33], MYB domain protein 36 (MYB36) [34], CASPs [35], GAPLESS [36], and dirigent proteins (DPs) [29]. Furthermore, the Casparian strip is crucial in mitigating abiotic and biotic stresses, including those induced by salinity, drought, and temperature fluctuations [37]. As one kind of stress, what is the relationship between the emergence of CCOs and the integrity of the Casparian strip? Could these Casparian strip-related genes respond to CCOs and play biological functions? These need further verification.

Small peptides, comprising 2 to 100 amino acid residues, are minuscule molecules that assume pivotal roles within living organisms. These molecules orchestrate plant growth, development, and environmental responses through a cascade of intricate signal transduction pathways. Small peptides are also called plant polypeptide hormones, given their similarities in function to traditional plant hormones [38]. Currently, known functional small peptides include CIF, Clavata 3/embryo surrounding region (CLE), Phytosulfokine (PSK), Inflorescence deficient in abscission (IDA), Plant elicitor peptide 1 (PEP1), and Rapid alkalinization factor (RALF). Among them, as SGN3/GSO1 and GSO2 ligands, the CIFs are required for diffusion barrier formation via regulating the formation of the Casparian strip in plants [39,40], which are extensively connected with various aspects of plant biology, including development, growth, immunity, and environmental adaptability. Moreover, five CIF members are expressed in different tissues and exert multiple biological functions in the *Arabidopsis* genome [41]. For example, *AtTWS1* is responsible for the deposition of the stratum corneum on epidermal cells and the organization of the intima system [42,43,44]. Pre-transcriptomic data revealed that *P. cablin PatCIF* small peptides could respond to the allelochemical p-HBA, implying that this gene family could be vital in combating the challenges of CCOs through its impact on the Casparian strip’s integrity [45]. However, how many members of the *PatCIF* family are in *P. cablin*? Which *PatCIF* genes could respond to the autotoxin p-HBA? Which members participate in regulating CCOs, and what are their signaling pathways? These questions require further investigation.

In this study, a comprehensive screening and identification of 12 members of the PatCIF family was conducted using bioinformatics technology based on the whole genome data of *P. cablin*. Subsequently, a detailed analysis was performed on the protein physicochemical assays, chromosomal localization, *cis*-acting elements promoter, and characteristics of evolutionary expression. At the same time, the *CIF* family of 33 species, including *S. lycopersicum*, *G. max*, and *O. sativa*, were compared, the possible evolutionary progress of the *CIF* family was predicted, and the collinearity relationship between *P. cablin* and *S. lycopersicum* or *O. sativa* was analyzed. Moreover, in-depth analysis of the transcriptome data of *P. cablin* at various phases of p-HBA treatment also helped to select and identify a total of 12 crucial *CIFs* and *GSO1* candidates engaged in CCOs. AlphaFold predicted the structure and combination pairing of CIFs and GSO1s. This study provides a crucial theoretical foundation for further investigation into the functions of the *PatCIF* gene family and their involvement in CCOs.

## 2. Results

### 2.1. Identification of PatCIF Genes and Their Physicochemical Characteristics

According to the five CIF protein sequences from *A. thaliana*, 12 potential *CIF* candidate genes were identified and screened within the *P. cablin* genome database through BLASTP alignment and subsequent deduplication processes. However, in the later transcriptome analysis, only 4 of the 12 *CIF* genes were identified to be related to the CCOs of *P. cablin*. The physicochemical characteristics of the 12 PatCIF proteins were predicted by the ExPASy online analysis tool (Table 1). The findings indicated that the amino acid count for the 12 members of the PatCIF family varied between 84 and 118. The gene encoding the fewest amino acids was PatTWS5, comprising just 84 amino acids, whereas the gene with the highest count was PatTWS7, consisting of 118 amino acids. The relative molecular mass of PatCIF protein ranged from 9.27 to 12.81 kDa, encompassing three acidic proteins (pI ≤ 7) and nine basic proteins (pI ≥ 7). The isoelectric point of 12 CIF proteins ranged from 5.60 to 9.51. The aliphatic index ranged from 58.10 to 99.55. Within the *CIF* gene family, the instability index for four *CIF* genes falls below 40, indicating stable proteins; conversely, eight *CIF* genes exhibit an instability index exceeding 40, signifying instability. The average hydrophilicity of the 12 proteins was below zero, indicating that they were all hydrophilic.

The secondary structure of PatCIFs protein was analyzed by SOPMA online software. The results showed that 10 genes in the PatCIF protein contained four types of structures: α-helix, β-turn, random coil, and extended chain, while PatTWS1 and PatTWS5 only had three types of structures: α-helix, random coil, and extended chain (Table 2). Among them, α-helix accounted for 9.52~47.19%, extended chain accounted for 4.49~20.54%, β-turn (except PatTWS1,5) accounted for 3.57~13.48%, and random coil accounted for 42.7~77.38%. It is worth noting that the α-helix structure appears in all PatCIF proteins, indicating that the α-helix structure plays an essential role in the function of the PatCIF protein, and the random coil structure also covers a large proportion of the PatCIF protein.

**Figure 1:**
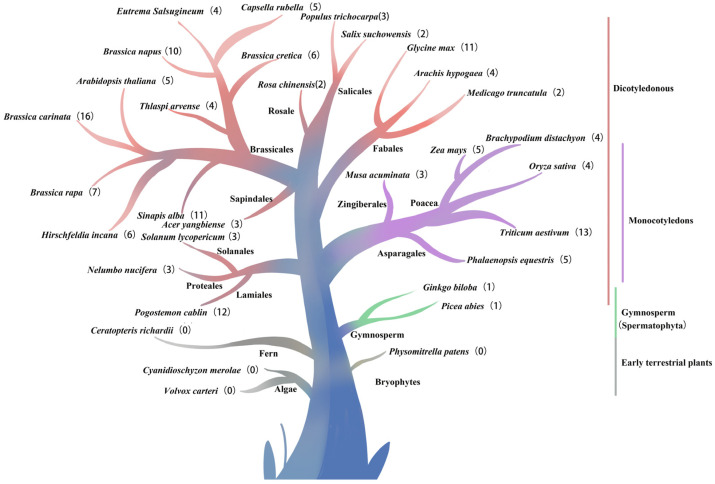
The illustrated relationships of the *CIF* gene family in 33 plant species. The numbers in parentheses indicate the number of *CIF* family members in each species. Red denotes the gene family associated with dicotyledonous plants in angiosperms, purple with monocotyledonous plants, green with the phylogenetic relationships associated with gymnosperms, and gray with the lower plants.

Through BLASTP of five AtCIF protein sequences in 33 species, homologous sequences were identified in 29 species (the specific number of pictures is shown in Figure 1). In lower plants and early terrestrial plants, the corresponding genes were not aligned in the algae *Volvox carteri* and *Cyanidioschyzon merolae*, the bryophyte *Physomitrella patens*, and the fern *Ceratopteris richardii*. Notably, a homologous gene was first identified in *Ginkgo biloba* and *Picea abies* in gymnosperm. In monocotyledonous plants, three homologous genes were identified in Zingiberales *Musa acuminata*, and five homologous genes of *the CIF* family were identified in Asparagales *Phalaenopsis equestris*. Four plants were identified in Poaceae, four homologous genes were recognized in *Brachypodium distachyon* and *O. sativa*, five homologous genes were identified in *Zea mays*, and thirteen homologous genes were identified in *Triticum aestivum*. In angiosperms, 2 homologous genes were identified in basal angiosperm *Amborella trichopoda*, 12 *CIF* family genes were identified in Lamiales *P. cablin*, 3 homologous genes were identified in Proteales *Nelumbo nucifera*, and 3 homologous genes were identified in Solanales *Solanum lycoperisum*. Three homologous genes were identified in Sapindales *Acer yangbiense*. The 10 species were identified in Brassicales, and 4 *CIF* family genes in *Thlaspi arvense* and *Eutrema Salsugineum*, 5 *CIF* family genes were identified in *Capsella rubella* and *A. thaliana*, 6 homologous genes in *B. cretica* and *Hirschfeldia incana*, 7 in *Brassica Rapa*, 10 in *B. napus*, 11 in *Sinapis Alba*, and 16 in *Brassica carinata*. In Salicales, two homologous genes were identified in *Salix suchowensis*, and three homologous genes were identified in *Populus trichocarpa*. Two homologous genes were identified in Rosales *Rosa chinensis*. In Fabales, two *CIF* homologous genes were identified in *Medicago truncatula*, four in *Arachis hypogaea*, and eleven in *G. max.* Upon investigation, it was discovered that the Cruciferae family exhibits a relatively high number of *CIF* gene families, and the phylogenetic tree analysis showed that the genetic relationship was relatively close. However, *CIF* gene homologous sequences were not identified in algae, ferns, and bryophytes. One *CIF* homologous sequence was identified in each of the gymnosperm spruce and ginkgo leaves, which belonged to the same branch as *AtTWS1*. It is inferred from Figure 1 that *CIF* originated from Spermatophyta, and the widespread existence of cross-homologous lineages proves that the *CIF* gene family may have originated before the separation of gymnosperms and angiosperms.

### 2.2. Analysis of Gene Characteristics and Chromosome Localization of PatCIF Genes

**Figure 2:**
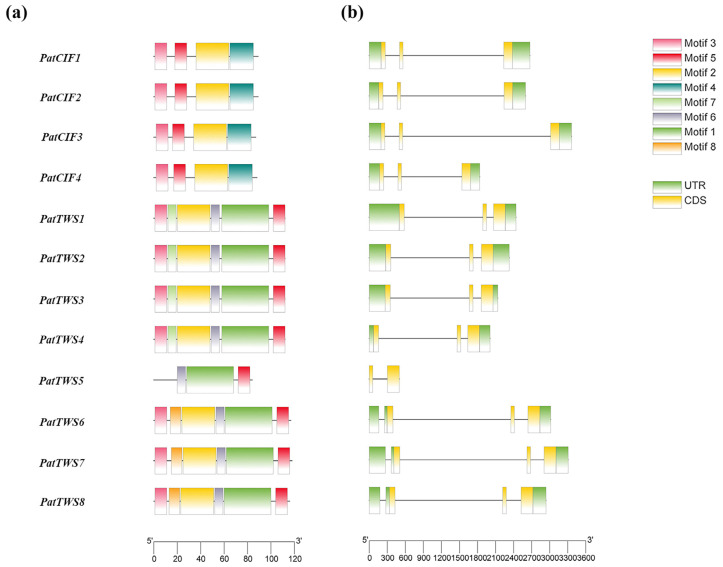
Conserved motifs (**a**) and gene structure (**b**) of *PatCIF* family members in *P. cablin.* (**a**) represents the exon-intron structure of *PatCIF*, and (**b**) shows the non-coding region (UTR) and coding sequence (CDS).

To further study the structural characteristics of the *PatCIF* genes, the Motif structure and gene structure of PatCIFs were visualized by MEME online tool and TBtools software (Figure 2). The results showed that the number of motifs in each member of the *PatCIF* gene family was not the same since *PatCIF1–4* had four conserved motifs, *PatTWS1–4* and *PatTWS6–8* contained six conserved motifs, and *PatTWS5* had only three conserved motifs (Figure 2a). According to the distribution of these conserved motifs, the *PatCIF* genes can be roughly divided into two groups: *PatCIFs* and *PatTWSs.* In addition, the members of the *PatCIF* gene family encompass two to six exons and one to five introns (Figure 2b).

**Figure 3:**
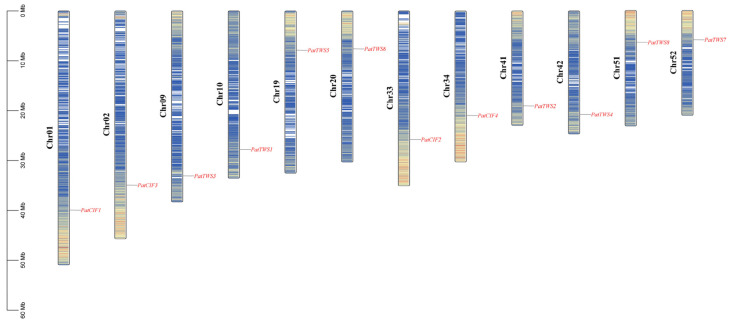
Chromosomal mapping of *PatCIF*s in *P. cablin*. The left scale 0–60 Mb represents the size of the chromosome; from left to right, Chr01 to Chr52 represents the names of 12 chromosomes in the *P. cablin* genome. Blue to red represent gene density from low to high, respectively.

Within the genome of *P. cablin*, 12 *CIF* genes were identified, each located on distinct chromosomes. By extracting the relevant data from the genome annotation file and utilizing the Tbtools for visualization, the distribution map of the *PatCIFs* and the genetic distance among genes were shown in Figure 3. In detail, *PatCIF1–4* is located on chromosomes 1, 33, 2, and 34, respectively. Moreover, *PatTWS1–4* is found on chromosomes 10, 41, 9, and 42, while *PatTWS5-8* is located on chromosomes 19, 20, 52, and 51, respectively.

### 2.3. Analysis of Cis-Acting Elements of the PatCIFs

**Figure 4:**
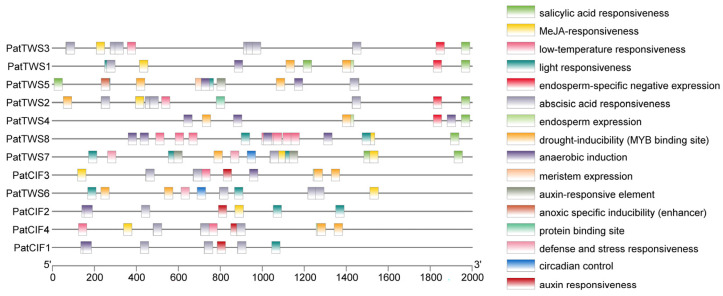
*Cis*-acting elements of *PatCIF* family members in *P. cablin.* Using the 2000 bp promoter region of the upstream sequence of the *PatCIF* gene, a range of *cis*-acting elements formed the right side of the figure.

To further study the PatCIFs-related signal pathways or networks, promoter 2000 base pairs (bps) upstream were selected and extracted to analyze *cis*-acting elements. The results indicated that 16 distinct types of *cis*-elements were identified within the promoter region of the *PatCIF* gene family. Among these elements, the number of light-responsive elements is the largest, followed by those responsive to abscisic acid, indicating that the *PatCIF* gene family shows a significant role in plant photomorphogenesis regulation and the stress response mediated by abscisic acid (Figure 4). Further analysis revealed that most *PatCIF* family members encompass auxin, gibberellin, methyl jasmonate, salicylic acid, other hormone response elements, and stress-related elements. In addition, some *cis*-elements related to the stress response pathway were found, such as anaerobic induction, low-temperature response, drought induction, and defense and stress response elements. It is worth noting that the *PatCIF* gene has a specific drought induction at the MYB binding site, which may mean that *PatCIF* can bind to and function with MYB under drought conditions. In addition, within the similar subcluster of the *PatCIF* gene family, the *cis*-regulatory elements of distinct genes exhibit variations, indicating that distinct *PatCIF* genes could assume diverse functions in growth, development, and stress responses. The findings suggest that the *PatCIF* gene family likely significantly regulates *P. cablin* growth and defense mechanisms.

### 2.4. Phylogenetic Analysis of the PatCIF Gene Family

**Figure 5:**
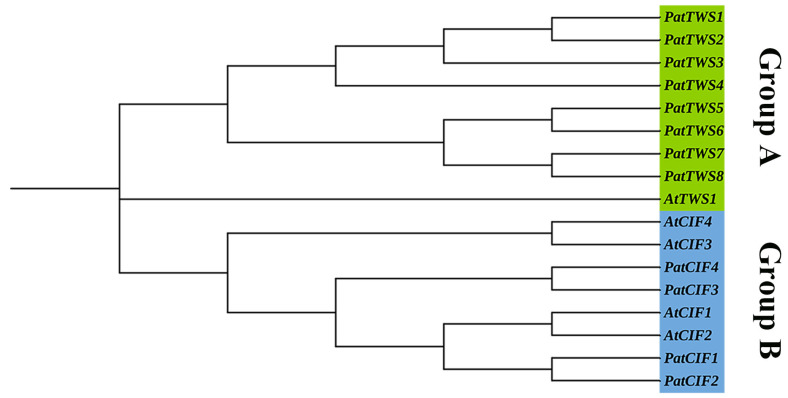
Groups A and B are two sub-clusters of Phylogenetic trees of *CIF* genes in *P. cablin* (Pat) and *A. thaliana* (At).

The phylogenetic relationships within the *PatCIF* gene family were subjected to further investigation. A phylogenetic tree was constructed for 17 CIF protein sequences from *P. cablin* and *Arabidopsis* using MEGA11 (Figure 5). The findings indicate that 17 members of the *PatCIF* and *AtCIF* can be distinctly categorized into two distinct subclusters, Group A and Group B, respectively. Group A consisted of one *Arabidopsis* gene and eight *P. cablin* genes, while Group B consisted of four *Arabidopsis* genes and four *P. cablin* genes. This suggests that the *CIF* genes within the *P. cablin* and *Arabidopsis* subcluster likely share a common ancestor and analogous functions. By comparing the *CIF* genes of *P. cablin* and *A. thaliana* in Figure 5, it was found that the *CIF* family may have chromosome doubling events in the *P. cablin* genome, resulting in increased *PatCIF* family genes within *P. cablin*, making it more numerous than other members. However, it is surprising that group A consists of one *A. thaliana* gene and eight *P. cablin* genes, all of which exhibit a doubling trend. This suggests that, in addition to chromosome doubling, other events may have influenced the doubling of the A subcluster during the evolution of *P. cablin*. This event may have a significant impact on the evolution of the *PatCIF* family.

### 2.5. Interspecific Collinearity Analysis of the PatCIF Gene Family

**Figure 6:**
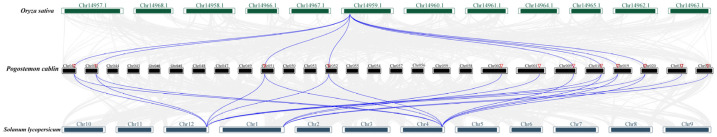
Collinearity analysis of *P. cablin* with species *O. sativa* or *S. lycopersicum*. The gray lines in the figure identify the collinearity region, while the blue lines indicate the *PatCIF* homologous gene pairs.

To further explore the evolutionary process of the *PatCIF* family, the collinearity analysis among species was performed. The dicotyledonous model plant, *S. lycopersicum*, and the monocotyledonous model plant, *O. sativa*, were chosen for pairwise analysis from the 29 species with homologous genes identified (Figure 6). The results showed 19 significant linear relationships between the *CIF* family of *P. cablin* and *S. lycopersicum* (Figure 6). In comparison, there were only eight linear relationships between the *CIF* family of *P. cablin* and *O. sativa*; it becomes apparent that *P. cablin*’s evolutionary ties to dicotyledonous plants are more intimate, which is expected that the collinearity is better conserved. The chromosomes of Chr10, Chr19, Chr30, Chr41, Chr42, Chr51, and Chr52 had more orthologous genes, and the collinearity was significantly stronger than other chromosomes. Seven genes, such as *PatTWS1-2* and *PatTWS4-8*, have at least two pairs of orthologous genes. These orthologous genes may come from common ancestral genes and may play similar roles in evolution. This coincides with the results of the phylogenetic analysis.

### 2.6. PatCIF Genes Expression Analysis

**Figure 7:**
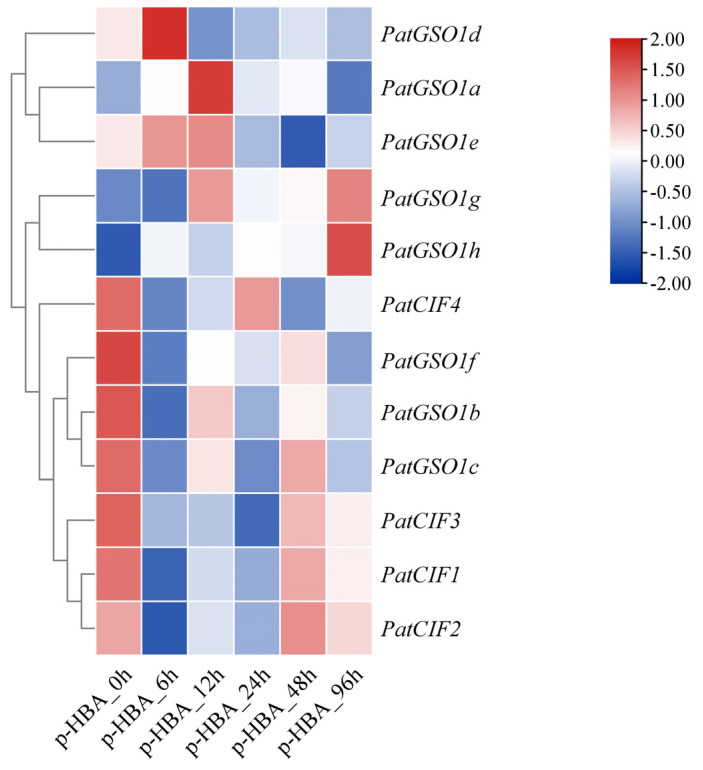
Expression of *PatCIF* and *PatGSO1* genes at different times treated by p-HBA. The ruler at the top right reflects the change in gene expression level, and its color gradually changes from red to blue, representing the transition from positive to negative.

In the previous analysis of *cis*-acting elements, it was found that the PatCIFs promoter region has many elements that respond to stress and various plant hormone signals, indicating that *PatCIFs* genes make a significant contribution to the stress regulation response mechanism of plants. To further explore the potential response mechanism of *P. cablin* under CCOs and detect the related response genes, *CIF* and *GSO1* genes associated with CCOs were screened out in the transcriptome data (NCBI Accession Number: PRJNA850618) [45]. The cluster heat map of the transcripts of four *CIF* genes and eight *GSO1* genes was constructed (Figure 7). The findings showed that the *CIF* expression and *GSO1* genes in the roots of *P. cablin*, in response to p-HBA, can be categorized into two groups: inhibited p-HBA and promoted expression p-HBA [46]. In the first group, relative to the 0 h, gene expression was markedly suppressed at 6 h, 12 h, or 24 h. Subsequently, the expression of certain genes was observed to increase at 48 h or 96 h, suggesting that p-HBA initially inhibited the expression of these genes, and its inhibition gradually diminished with the passage of time or p-HBA consumption. Seven genes, including *PatCIF1*, *PatCIF2*, *PatCIF3*, *PatCIF4*, *PatGSO1b*, *PatGSO1c*, and *PatGSO1f*, are in this type. In the second group, p-HBA treatment can stimulate the expression of *PatGSO1a*, *PatGSO1d*, *PatGSO1e*, *PatGSO1h*, and *PatGSO1g* compared to the 0 h, albeit with varying response times. In detail, the gene expression of *PatGSO1d* was higher at 6 h and then declined. The expression levels of the genes *PatGSO1a*, *PatGSO1g*, and *PatGSO1e* peaked at 12 h. The *PatGSO1h* gene expression was substantially down-regulated at 96 h. Remarkably, the genes *PatCIF1* and *PatCIF2* were highly expressed before p-HBA treatment and dramatically decreased at 6 h, implying that p-HBA significantly affects the expression of two key genes and Casparian strip integrity under CC conditions. The marked variations in the upregulation and downregulation of these genes could disrupt the normal development of the Casparian strip, compromise the resilience of *P. cablin* against adversity, and ultimately result in the occurrence of CCOs. Nevertheless, these genes’ precise functions and mechanisms require additional experimental validation and thorough investigation.

### 2.7. Analysis of Tertiary Structure and Protein Interaction Between PatCIFs and PatGSO1s

**Figure 8:**
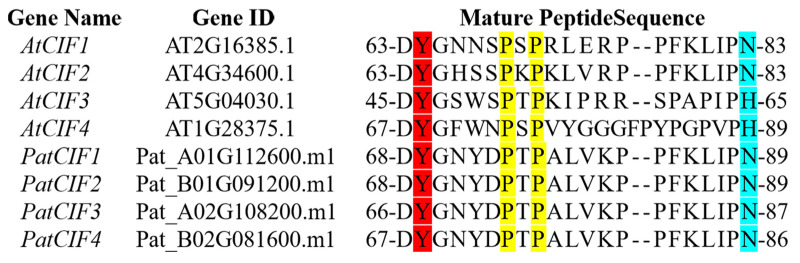
Multiple sequence alignments of *CIFs* in *P. cablin* and *A. thaliana* (The conserved sTyr is highlighted in red, the hydroxyproline is highlighted in yellow, and the C-terminal asparagine/histidine is shown in blue).

**Figure 9:**
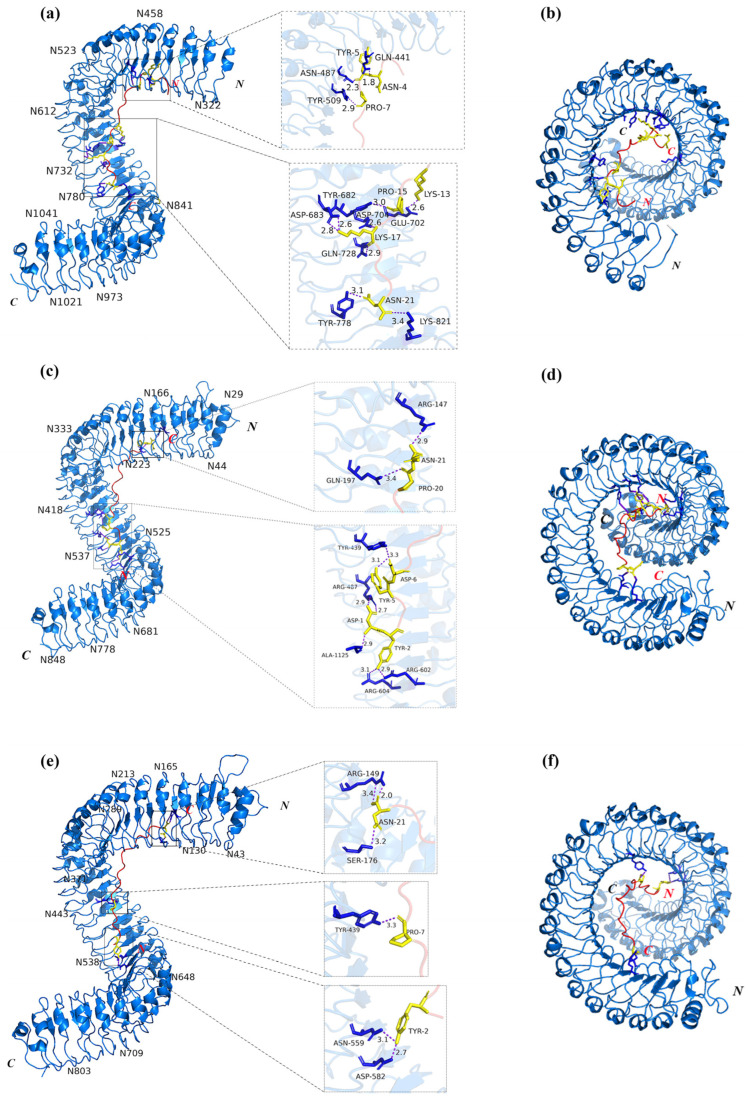
PatCIF mature peptide and *PatGSO1b*, *PatGSO1c*, *PatGSO1f* protein interaction prediction map. (**a**,**c**,**e**) is the main view of the binding diagram of *PatGSO1b* and PatCIF mature peptide, *PatGSO1c* and PatCIF mature peptide, *PatGSO1f* and PatCIF mature peptide; (**b**,**d**,**f**) is the top view of the binding map of *PatGSO1b* and PatCIF mature peptide, *PatGSO1c* and PatCIF mature peptide binding map, *PatGSO1f* and PatCIF mature peptide binding map. In (**a**,**c**,**e**), blue is *PatGSO1S* binding sticks, yellow is PatCIF mature peptide binding sticks, black font is the name of the binding site, purple is the binding hydrogen bond, and the number is the length of the binding hydrogen bond.

To investigate the interaction between *PatCIFs* and *PatGSO1s*, four *PatCIFs* expressed in the transcriptome were first subjected to multiple sequence alignment with four *AtCIF* genes of *A. thaliana* (Figure 8) [47] based on the similar mature peptide structure. The results showed the N-terminal of PatCIFs mature peptide contains a conserved DY motif [48,49]. Meanwhile, there is a hydroxyproline motif in the middle and asparagine/histidine in the C-terminal. Interestingly, the mature peptide sequence of *PatCIF1–4* was consistent by comparison. Subsequently, three downregulated *PatGSO1s* genes (*PatGSO1b*, *PatGSO1c*, and *PatGSO1f*) screened from transcriptome data were selected for further interaction analysis. AlphaFold3 predicted mature peptide protein sequences in PatGSO1s and PatCIF1–4, and the model with the highest confidence in the prediction was chosen to construct a protein interaction map. Combined with NCBI and InterPro domain analysis, the helical LRR structure was selected for composition analysis (Figure 9). The outer domain of *PatGSO1s* contains LRR folded into a super-helical assembly form. It can be seen on the graph that the structure has about 1.5 helix rotations. This domain provides a key binding surface for mature PatCIF peptides. The *PatGSO1b* and 21 amino acids are predicted to form 11 hydrogen bonds, of which LYS-17 and ASP-683 form two hydrogen bonds. Nine hydrogen bonds were formed between *PatGSO1c* and the mature peptide. The ASP-1 and ARG-487 formed two hydrogen bonds. *PatGSO1f* formed six hydrogen bonds, and there were two hydrogen bonds between ASN-21 and ARG-149, further ensuring the realization of the interaction function. It is worth noting that the conserved sTyr at the N-terminus of mature PatCIF interacts with the LRR domain at the N-terminus of PatGSO1b in the prediction but interacts with the C-terminus of PatGSO1c and PatGSO1f. It is speculated that the LRR domain of PatGSO1b is mainly distributed at the N-terminus, while *PatGSO1c* and *PatGSO1f* are primarily distributed at the C-terminus (Figure S1), which in turn affects the interaction sites. The prediction results verified the signaling pathways of *PatCIF* and *PatGSO1* to a certain extent, which laid a solid theoretical foundation for the further study of the biological functions of *PatCIF* and *PatGSO1*.

### 2.8. qRT-PCR Validation

**Figure 10:**
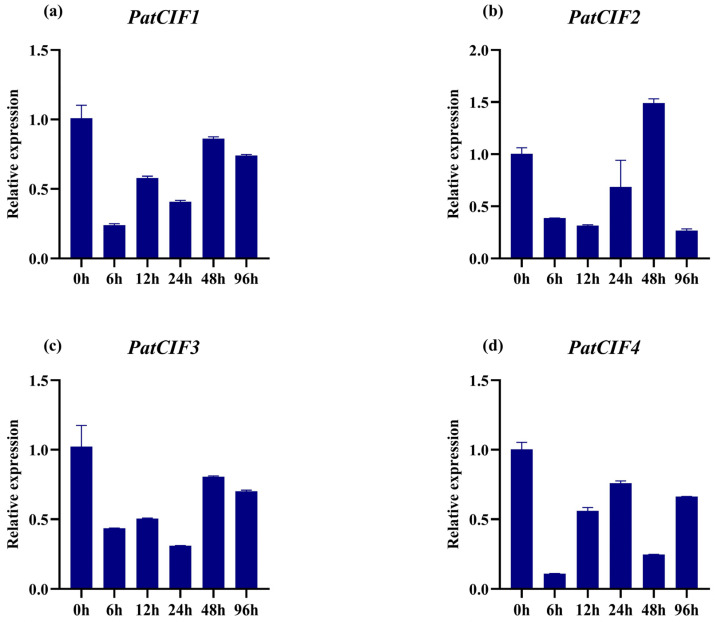
Expression levels of *PatCIFs* genes at 0 h, 6 h, and 12 h under p-HBA treatment. (**a**) *PatCIF1*; (**b**) *PatCIF2*; (**c**) *PatCIF3*; (**d**) *PatCIF4*.

In order to further verify the function of *PatCIF* genes in response to abiotic stress, four *CIF* genes (*PatCIF1–4*) were selected as candidate genes for response to p-HBA according to the screening results of transcriptome data (Figure 10). The roots of *P. cablin* seedlings under 1 mmol/L stress at 0 h, 6 h, and 12 h were detected and analyzed by qRT-PCR. From Figure 9, it can be seen that under p-HBA stress, all *PatCIF* genes showed a downward trend and decreased expression. Among them, *PatCIF1* was rapidly downregulated at 6 h, reached the lowest point, and then slowly increased; *PatCIF2* was downregulated at 6 h and 12 h, increased, reached the highest point at 48 h, and decreased. *PatCIF3* showed a downward trend and reached the lowest point at 24 h. The *PatCIF4* was rapidly downregulated after treatment and reached the lowest point at 6 h. The downregulation of these genes shows that p-HBA initially inhibited their expression, and the inhibitory effect was weakened to varying degrees over time, as well as the consumption of p-HBA. These results indicate that *PatCIFs* can respond to p-HBA treatment and may play an important role in responding to challenges related to CCOs.

## 3. Discussion

As a key gene regulating the barrier in the Casparian strip, CIF affects the absorption of mineral elements and the integrity of the Casparian strip and regulates tapetum development and pollen wall formation [48]. The 12 *CIF* genes were successfully identified using bioinformatics analysis. In addition to *P. cablin*, *CIF* genes were also found in 29 different species (Figure 1), indicating that this gene family has a wide distribution and important functions in the plant kingdom. Via the analysis of these *CIF* genes, there are relatively more homologous genes in *B. napus*, *S. Alba*, *G. max*, *P. cablin*, *B. carinata*, and *T. aestivum* since most of them are tetraploid, and *T. aestivum* is hexaploid [50]. About 1.1 million years ago, the genome of *P. cablin* experienced an LTR-RT insertion event, which may further enrich the species and number of *CIF* genes [49]. Genome duplication events, tandem repeats, and natural selection are likely to shape the diversity of *CIF* gene families to varying degrees.

This study found that CIF homologous genes in lower and early terrestrial plants were not found in algae, bryophytes, and ferns. However, one homologous gene was identified in gymnosperms and expanded on a large scale in angiosperms (Figure 1). Previous research has indicated that the Casparian strip is present in all vascular plants, encompassing lycophytes, angiosperms, ferns, and gymnosperms. In contrast, suberin lamellae are found exclusively in seed plants, which include gymnosperms and angiosperms [51]. Therefore, it is speculated that the *CIF* gene may originate together with genes associated with suberin layer formation; the *CIF* gene finely regulates the biological function of the Casparian strip, thereby promoting seed plants to better adapt to the terrestrial environment.

*Cis*-element analysis revealed that the *PatCIF* gene family was rich in hormone and stress response factors (Figure 4). These elements indicate that *PatCIFs* could be pivotal in plants’ growth, development, and stress response. Studies have shown that in other species, such as *Arabidopsis*, the *CIF* gene is involved in a variety of biological processes, including maintaining the integrity of the Casparian strip, tapetum development, pollen wall formation [48], and embryonic epidermis formation [52]. These findings further corroborate the perspective that *PatCIF* genes also play a pivotal role in growth, developmental regulation, and stress response. Notably, in *Arabidopsis*, *AtCIF1/2* is mainly expressed in the root stele. Under low potassium conditions, K^+^ in the xylem of *cif1cif2* mutant is relatively reduced, indicating that *AtCIF1/2* has an essential effect on K^+^ uptake and accumulation in *Arabidopsis* [31]. In addition, the study found that under salt stress, the Casparian strip-related gene *GSO1* can effectively inhibit the entry of Na^+^ ions into the vascular tissue system and avoid potential damage to the fragile stem cells in the meristem. This mechanism of action is achieved by protecting the integrity of the meristem, thereby activating the SOS2-SOS1 module mediated by the receptor-like kinase to ensure that the root system can still maintain its growth function in harsh environments [32]. The biological function of the *P. cablin PatCIF* gene family requires further experimental verification.

The collinearity analysis between *P. cablin*, *O. sativa*, and *S. lycopersicum* found 19 and 8 collinearity relationships between *P. cablin* and *S. lycopersicum* or *O. sativa*, respectively. These collinearity relationships suggest that the *PatCIF* family of *P. cablin* may share some key gene arrangements and functions with *S. lycopersicum* and *O. sativa* during the long evolutionary process. This sharing may be derived from their common ancestors or through mechanisms such as horizontal gene transfer during evolution. This finding helps to understand the evolutionary process of the *PatCIF* family in *P. cablin*. It provides a new perspective for studying the genetic relationship between *P. cablin*, *S. lycopersicum*, and *O. sativa*. In addition, through transcriptome and qRT-PCR analysis, we observed that the *PatCIF* gene showed a downward trend, indicating that p-HBA significantly affected the expression of *CIF* key genes under continuous cropping conditions. The significant downregulation of these gene expressions may interfere with the normal development of the Casparian strip, weaken the resistance of *P. cablin* to adversity, and ultimately lead to the occurrence of CCOs.

As a kind of traditional medical plant, *P. cablin* is faced with the problem of CCOs in production practice, which seriously restricts the sustainable development of its industry. Due to long-term continuous planting on the same land, CCOs frequently result in alterations to soil physical and chemical properties, shifts in the structure of microorganisms, exacerbation of soil-borne diseases, and significant allelopathic autotoxicity [53,54]. Previous research has established that p-HBA is the primary allelochemical responsible for the CCOs of *P. cablin.*

In this study, the transcriptome data of p-HBA-treated *P. cablin* roots were intensely mined, and 12 potential p-HBA stress-responsive genes, namely PatCIFs and PatGSO1s, were successfully identified. These genes showed different expression patterns under p-HBA stress. Among them, *PatCIF1–4*, *PatGSO1b*, *PatGSO1c*, and *PatGSO1f* expressions were downregulated. This alteration in expression could disrupt the typical development of the Casparian strip, potentially diminishing *P. cablin* resilience to stress. Further, this phenomenon could result in the appearance of CCOs and adversely affect the growth and development of *P. cablin*. Moreover, this allows the allelochemicals to enter the vascular bundle via the incomplete Casparian strip and carry out long-distance transportation, thereby seriously disrupting plant metabolism, affecting plant growth and development, and ultimately causing CCOs of *P. cablin*. At the same time, the expression of other *PatGSO1* genes was upregulated, potentially restoring the integrity of the Casparian strip to some degree and decelerating the penetration of allelochemicals into the plant’s vascular bundles. The findings offer a novel perspective and potential solution for understanding the molecular mechanism behind *P. cablin*’s response to CCOs. Nevertheless, the precise roles of these candidate genes remain to be studied and confirmed in greater detail.

## 4. Materials and Methods

### 4.1. Genome-Wide CIF Identification in P. cablin

*A. thaliana* genomic data were reliably received from the TAIR (https://www.arabidopsis.org/), the genome sequence file, protein sequence, and gene structure annotation file for *P. cablin* from the GSA (https://ngdc.cncb.ac.cn/gsa/, accessed on 14 May 2024) (accession number: CRA004172) [49]. Five CIF proteins from *A. thaliana* have extracted amino acid sequences using TBtools (v2.084) [55]. The *G. max*, *C. richardii* genome data were downloaded from the phytozome website (https://phytozome-next.jgi.doe.gov/), the wheat genome data were downloaded from the wheat gene network (https://www.wheatgenome.org/), the *Populus trichocarpa*, *S. lycoperisum*, *P. patens* were downloaded from the ensemble website, the *G. biloba* genome was downloaded on the *G. biloba* gene network (http://gigadb.org/dataset/100613#, accessed on 14 May 2024), and the genome data of *R. chinensis*, *A. hypogaea*, *O. sativa*, and other species were downloaded on the NCBI website (https://www.ncbi.nlm.nih.gov/).

The *Arabidopsis CIF* gene protein sequence was aligned with the *P. cablin* protein data (e^−value^ < 1 × 10^5^) using BLASTP to obtain homologous sequences and then remove duplicates. The protein sequence of the *A. thaliana CIF* gene was compared by BLASTP in the *Picea abies* gene network (https://plantgenie.org/).

The Expasy website (https://www.expasy.org/) was utilized to predict the amino acid count and protein molecular weight of CIF family members, employing the SOPMA online software (https://npsa-prabi.ibcp.fr/, accessed on 14 May 2024) to predict and analyze the secondary structure of CIF family proteins.

### 4.2. Analysis of Gene Characteristics and Chromosomal Localization of the CIF Small Peptide Family

The position data for UTR, exons, and introns within the *CIF* family genes was derived from the *P. cablin* genome annotation (gff) file, and a structural map of the *CIF* genes was generated utilizing TBtools software. The MEME [56] online (http://meme-suite.org/) was used to analyze the conserved motifs of CIF proteins. The motifs reached a maximum of 8 in number, with lengths ranging from 6 to 50 amino acids. For visual analysis, TBtools software was employed.

The genome annotation file for *P. cablin* provides detailed information on the lengths of the plant’s 12 chromosomes and the precise locations of all *PatCIF* genes on these chromosomes. With position information and distance connections of all *P. cablin PatCIF* gene family members on chromosomes highlighted, the TBtools program was used to visualize the chromosomal location map of the *P. cablin PatCIF* gene.

### 4.3. Cis-Elements Analysis of CIF Gene Family Members in P. cablin

The promoter sequences for the *PatCIF* genes were obtained 2000 bp upstream of the transcription start site using TBtools. The PlantCARE (http://bioinformatics.psb.ugent.be/webtools/plantcare/html/, accessed on 16 May 2024) then helped to find the *cis*-regulatory elements inside the *PatCIF* gene’s promoter region and visualized by Tbtools [57].

### 4.4. CIF Gene Family Members’ Phylogenetic Analysis in P. cablin

The CIF protein sequence of *P. cablin* and *A. thaliana* was compared using a muscle program, and the outcomes of this comparison were employed to predict the optimal model via ProtTest 3 [58]. MEGA11 software [59], using the maximum likelihood approach, built the phylogenetic tree of CIF protein in *P. cablin* and *Arabidopsis*; the CIF protein sequences found in 29 species were compared using the Neighbor Joining (NJ) technique and then visualized by ITOL online tool (https://itol.embl.de/, accessed on 15 May 2024).

### 4.5. CIF Gene Family Members’ Interspecific Collinearity Analysis in P. cablin

The homology of *CIF* family members of *P. cablin*, *S. lycopersicum*, and *O. sativa* was analyzed using the MCScanX [60]. Subsequently, TBtools software [55] was used to generate a collinearity analysis diagram to visually display the genetic relationship of *CIF* family members among species.

### 4.6. PatCIF Gene Family Members’ Expression Study in P. cablin

Based on the preceding transcriptomic analysis of *P. cablin* roots, transcriptomic data were acquired for *P. cablin* roots at various time intervals (0 h, 6 h, 12 h, 24 h, 48 h, and 96 h) following exposure to p-HBA. The raw data mentioned above have been uploaded to the NCBI (https://www.ncbi.nlm.nih.gov/) SRA (sequence read file) with the “PRJNA850618” accession number.

The transcriptomic data pertinent to the *CIF* gene were obtained from the aforementioned transcriptome dataset and were initially screened by Microsoft Office 2019’s Excel software. The data were subsequently processed using TBtools software (v2.084), and the clustering heat map was generated.

### 4.7. RNA Extraction, cDNA Synthesis, and qRT-PCR Analysis in P. cablin

To analyze the *PatCIFs* gene expression pattern, *P. cablin* seedlings were treated with 1 mmol/L p-HBA stress. RNA extraction follows the instructions provided in the Plant RNA Extraction Kit (DP452, Tiangen, Beijing, China). The cDNA Synthesis Kit (MR05101, Monad, Suzhou, China) Reverse transcription of extracted RNA into cDNA. Four *CIF* genes were amplified using PerfectStart Green qPCR SuperrMix (AQ601, TransGen, Beijing, China). The 0 h, 6 h, and 12 h cDNA were used as templates, and *18SrRNA* was used as an internal reference gene. The primer pairs used for qRT-PCR analysis are shown in Table 3. Three replicates were set for each sample. After the reaction, the expression levels of four *CIF* genes were calculated by the 2^−ΔΔCT^ method.

### 4.8. The Tertiary Protein Structure and Protein Interaction Analysis of the PatCIFs and PatGSO1s in P. cablin

High-expressed *PatCIFs* and *PatGSO1s* were selected for further analysis based on transcriptome data. The protein sequences of the selected genes were retrieved from the genome file, and multiple sequence alignments were performed. The tertiary structure of *PatGSO1s* and the interaction between *PatGSO1s* and mature peptides in *PatCIFs* were predicted on the AlphaFold Server [61] online website (https://golgi.sandbox.google.com/). Combined with NCBI Conserved Domains and InterPro domain prediction, the data were further processed in PyMol (v3.0) software, and the interaction diagram was drawn.

## 5. Conclusions

*P. cablin* is a traditional southern medicine, spice, and medicine food homology plant in China, which holds significant pharmaceutical and commercial value. However, CCOs often restrict yield and quality. The *CIF* is a pivotal gene responsible for the development and maintenance of the Casparian strip, playing a crucial role in plant stress responses. In this research, the *CIF* gene family of *P. cablin* was systematically screened and identified at the genome-wide level. The characteristics of the family members, such as composition structure, physical and chemical properties, and evolutionary relationships, were analyzed. The distribution and evolution of the *CIF* gene family in different species and its potential functions in plant growth, development regulation, and stress response were preliminarily explored. Furthermore, by analyzing the transcriptome data, a series of potential stress response genes closely related to the CCOs of *P. cablin* were excavated, and qRT-PCR experiments verified that the gene expressions of *PatCIFs* were inhibited under p-HBA induction. AlphaFold3 predicted the mature peptide CIFs-GSO1s protein interaction of *P. cablin*. The results of this study laid a theoretical foundation for further analysis of the biological function of *PatCIFs* and their roles in CCOs. They provided a new idea for solving the problem of CCOs of *P. cablin.* However, the biological functions of these genes need further experimental verification. Future studies will focus on verifying the biological functions of these genes in different development, growth, and stress of *P. cablin* to reveal their contribution to species evolution and environmental adaptation.

## Figures and Tables

**Table 1:** *PatCIF* gene family physicochemical characteristics in *P. cablin*, including gene ID, amino acids, molecular weight, pI, instability index, aliphatic index, and GRAVY.

Gene ID	Gene Name	Amino Acids	Molecular Weight (kDa)	Theoretical pI	Instability Index	Aliphatic Index	GRAVY
Pat_A01G112600.m1	*PatCIF1*	89	10.17	6.73	39.54	99.55	−0.20
Pat_B01G091200.m1	*PatCIF2*	89	10.15	5.60	39.54	99.55	−0.19
Pat_A02G108200.m1	*PatCIF3*	87	9.89	6.73	38.49	94.02	−0.34
Pat_B02G081600.m1	*PatCIF4*	88	9.97	8.01	35.98	94.09	−0.24
Pat_A10G111700.m1	*PatTWS1*	112	11.88	9.51	42.00	79.20	−0.15
Pat_B09G103800.m1	*PatTWS2*	112	11.87	9.51	41.71	78.30	−0.16
Pat_A09G128900.m1	*PatTWS3*	112	11.90	9.51	41.71	80.00	−0.14
Pat_B10G099600.m1	*PatTWS4*	112	11.89	9.51	43.05	79.20	−0.16
Pat_A19G079100.m1	*PatTWS5*	84	9.27	8.09	61.47	58.10	−0.88
Pat_A20G075400.m1	*PatTWS6*	117	12.65	9.05	41.81	75.04	−0.39
Pat_B20G071600.m1	*PatTWS7*	118	12.81	9.05	43.17	74.41	−0.37
Pat_B19G076300.m1	*PatTWS8*	116	12.71	9.05	47.58	69.83	−0.44

**Table 2:** Secondary structure analysis of *PatCIF* gene family in *P. cablin*, including Alpha helix, extended strand, Beta turn, and random oil.

Gene Name	Alpha Helix%	Extended Strand%	Beta Turn%	Random Oil%
*PatCIF1*	47.19	4.49	5.62	42.70
*PatCIF2*	31.46	11.24	13.48	43.82
*PatCIF3*	40.23	9.20	5.75	44.83
*PatCIF4*	44.32	9.09	1.14	45.45
*PatTWS1*	20.54	14.29	0.00	65.18
*PatTWS2*	10.71	20.54	3.57	65.18
*PatTWS3*	14.37	19.38	9.38	56.88
*PatTWS4*	11.61	19.64	0.89	67.86
*PatTWS5*	9.52	13.10	0.00	77.38
*PatTWS6*	20.51	11.97	5.13	62.39
*PatTWS7*	13.56	20.34	8.47	57.63
*PatTWS8*	16.38	17.24	3.45	62.93

**Table 3:** Primer sequences of *PatCIF1–4* utilized in quantitative reverse transcription polymerase chain reaction (qRT-PCR) experiments.

Gene Name	Forward Primer Sequence (5′ → 3′)	Reverse Primer Sequence (5′ → 3′)
*PatCIF1*	GCTACCAGTGGTGTCAAAGGA	AGGTGGCTTAACGAGTGCTG
*PatCIF2*	AGCTACCAGTGGTGTCAAATGA	AGTGCTGGCGTAGGATCATAAT
*PatCIF3*	CTTCTGAAGGTCGGAGAGTGAAT	TGGCGTAGGATCATAATTCCCA
*PatCIF4*	TTCTTTTGCAGGTCGGAGAGT	AGTGCTGGCGTAGGATCATAAT
*18S rRNA*	CCGACCATAAACGATGCCGACC	TTTCAGCTTTGCAACCATACTCC

## Data Availability

The data presented in this study are openly available in NCBI at https://www.ncbi.nlm.nih.gov/bioproject/PRJNA850618/, accessed on 11 May 2024, reference number PRJNA850618.

## Supplementary Materials

The following supporting information can be downloaded at https://www.mdpi.com/article/10.3390/ijms26041568/s1.
