# Supplementary material for: Genome-Wide Identification of the CIF Gene Family and Protein Interaction with GSO1s Under the p-HBA-Induced Continuous Cropping Obstacle in Pogostemon cablin
Source: Int J Mol Sci. 2025 Feb 13;26(4):1568. doi: 10.3390/ijms26041568 (PMC11855783; doi:10.3390/ijms26041568)
Supplement: Supplementary file 1 [file ijms-26-01568-s001.zip › ijms-3204209-supplementary.docx]

**Supplementary Figure**


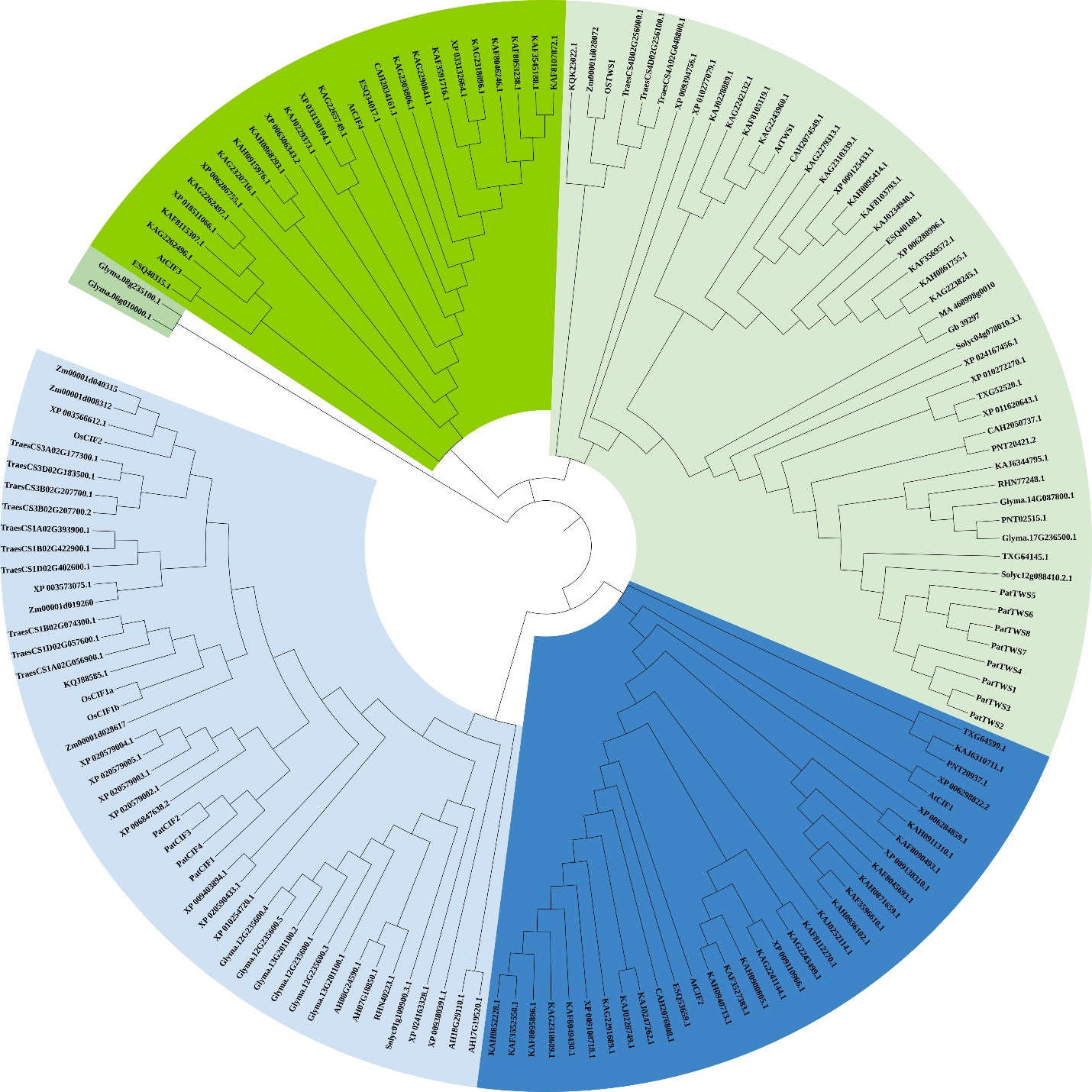


**Figure S1.** The phylogenetic relationships of the *CIF* gene family in 33 plant species.
